# Disrupted Gamma Synchrony after Mild Traumatic Brain Injury and Its Correlation with White Matter Abnormality

**DOI:** 10.3389/fneur.2017.00571

**Published:** 2017-10-30

**Authors:** Chao Wang, Michelle E. Costanzo, Paul E. Rapp, David Darmon, Dominic E. Nathan, Kylee Bashirelahi, Dzung L. Pham, Michael J. Roy, David O. Keyser

**Affiliations:** ^1^Traumatic Injury Research Program, Department of Military and Emergency Medicine, Uniformed Services University of the Health Sciences, Bethesda, MD, United States; ^2^The Henry M. Jackson Foundation for the Advancement of Military Medicine, Inc., Bethesda, MD, United States; ^3^Department of Medicine, Uniformed Services University of the Health Sciences, Bethesda, MD, United States; ^4^Graduate School of Nursing, Uniformed Services University of the Health Sciences, Bethesda, MD, United States; ^5^Center for Neuroscience and Regenerative Medicine, The Henry M. Jackson Foundation for the Advancement of Military Medicine, Inc., Bethesda, MD, United States

**Keywords:** mild traumatic brain injury, EEG, neural synchronization, functional connectivity, white matter integrity

## Abstract

Mild traumatic brain injury (mTBI) has been firmly associated with disrupted white matter integrity due to induced white matter damage and degeneration. However, comparatively less is known about the changes of the intrinsic functional connectivity mediated *via* neural synchronization in the brain after mTBI. Moreover, despite the presumed link between structural and functional connectivity, no existing studies in mTBI have demonstrated clear association between the structural abnormality of white matter axons and the disruption of neural synchronization. To investigate these questions, we recorded resting state EEG and diffusion tensor imaging (DTI) from a cohort of military service members. A newly developed synchronization measure, the weighted phase lag index was applied on the EEG data for estimating neural synchronization. Fractional anisotropy was computed from the DTI data for estimating white matter integrity. Fifteen service members with a history of mTBI within the past 3 years were compared to 22 demographically similar controls who reported no history of head injury. We observed that synchronization at low-gamma frequency band (25–40 Hz) across scalp regions was significantly decreased in mTBI cases compared with controls. The synchronization in theta (4–7 Hz), alpha (8–13 Hz), and beta (15–23 Hz) frequency bands were not significantly different between the two groups. In addition, we found that across mTBI cases, the disrupted synchronization at low-gamma frequency was significantly correlated with the white matter integrity of the inferior cerebellar peduncle, which was also significantly reduced in the mTBI group. These findings demonstrate an initial correlation between the impairment of white matter integrity and alterations in EEG synchronization in the brain after mTBI. The results also suggest that disruption of intrinsic neural synchronization at low-gamma frequency may be a characteristic functional pathology following mTBI and may prove useful for developing better methods of diagnosis and treatment.

## Introduction

Traumatic brain injury (TBI) is a major health challenge in both civilian and military populations (1, 2). Mild TBI (mTBI) is estimated to account for over 85% of all TBI cases (3). Specifically, mTBI has been recognized as a signature wound of the wars in Iraq and Afghanistan (4). According to surveys (5–7), approximately 15–23% of returning soldiers suffer from mTBI. Following an mTBI individuals often experience difficulties in cognitive functions such as memory, executive function and processing speed (8, 9), resulting in a significant negative impact on quality of life. Despite decades of research, the exact pathophysiology underlying the cognitive sequelae of mTBI remains unclear.

Cognitive functions rely on the integration of distributed processing of neuronal groups in large-scale brain networks (10–12). Structurally, the distributed neuronal groups, or the so-called “nodes” in the brain network, are connected to each other through white matter axons. Recent studies using advanced imaging techniques such as diffusion tensor imaging (DTI) have identified diffuse axonal injury, which likely represents selective damage of white matter tracts due to acceleration and deceleration forces or explosive blasts, as a major neuropathological consequence of mTBI (13, 14). This disruption in structural connectivity is expected to affect functional connectivity of brain networks, leading to network dysfunction and cognitive impairments (15).

In contrast with a substantial prior literature showing the impairments of structural connectivity in mTBI, little is known about the effect of mTBI on functional connectivity. It is widely understood that functionally, the coordination and communication between the distributed neuronal groups are mediated *via* synchronization of neural oscillations (16–18). Most previous work investigated the network dysfunction after mTBI by analyzing blood-oxygen-level-dependent signals [e.g., Ref. (19, 20)], which is an approximate and indirect measure of neural activity. Only a handful of studies (21–24) have specifically examined synchronization from electrophysiological data (EEG and MEG). In addition, despite the presumed link between structural and functional connectivity, no studies have yet demonstrated an association between the structural abnormality of white matter tracts and the disruption of neural oscillatory synchronization in any forms of TBI. Bridging this gap is important for further understanding of the pathophysiology of the disorder and may lead to better methods of diagnosis and treatment.

Methodologically, within the small number of the prior studies in mTBI that have examined synchronization using EEG or MEG, most of them employed conventional measures of synchronization such as coherence and phase locking value (PLV). It is known that these conventional measures are sensitive to the problem of volume conduction (25, 26). Nearby scalp electrodes or sensors are very likely to pick up activity from the same cortical sources, leading to spurious associations between the time series from these electrodes or sensors. Several alternative measures of synchronization including imaginary component of the coherency (ImC) (27), phase lag index (PLI) (26), and weighted phase lag index (WPLI) (28) have been introduced to deal with this problem. These measures are based upon the observation that non-zero-lag phase synchronization cannot be generated by volume-conducted activity from uncorrelated common sources, and are therefore likely to reflect true underlying brain connectivity. Among these measures the WPLI was developed most recently. Compared to PLI and ImC, WPLI has been demonstrated to be less sensitive to additive noise and has higher statistical power to detect changes in phase synchronization (28). Applying WPLI to study the abnormality of neural synchronization in mTBI may provide new insights into the problem.

In this study, we applied the WPLI analysis to resting EEG data recorded from military service members within two months of their return from a deployment in either Iraq or Afghanistan. Fifteen service members with a history of mTBI within the past 3 years were compared to 22 service members with no history of head injury from the same cohort. The association between neural synchronization and white matter integrity was also examined. We hypothesize that the intrinsic functional connectivity mediated *via* phase synchronization of neural oscillations will be affected after mTBI and the synchronization abnormality will be associated with white matter structural abnormality.

## Materials and Methods

### Participants and Psychological Assessments

Fifteen individuals with mTBI occurring in the past 3 years (mTBI group, 12 men, 3 women, mean age 27.1 ± 6.2 years) and 22 individuals with no history of head injury (control group, 21 men, 1 women, mean age 27.9 ± 6.1 years) were included in this study for comparisons. Both mTBI and controls were from the same cohort of military service members within 2 months of their return from a deployment in either Iraq or Afghanistan. The diagnosis of mTBI was determined by the Military Acute Concussion Evaluation (29). Most of the mTBI participants reported multiple head injury events. The types of head injury include combat-related blast exposure (*n* = 8), motor vehicle accident (*n* = 6), sports injury (*n* = 5), and accidental fall (*n* = 3).

All participants were administrated a series of validated questionnaires including the PTSD Checklist-Military Version (PCL-M), a self-administered screen for PTSD (30), the Patient Health Questionnaire-9 (PHQ-9), a widely used instrument for screening depression (31), and the Pittsburgh Sleep Quality Index (PSQI), a self-report instrument for assessing sleep over the prior month (32). Inclusion in the study required a score of less than 10 on the PHQ-9 and less than 50 on the PCL-M. In addition, an experienced, licensed psychologist conducted the Clinician-Administered PTSD Scale for DSM-IV (CAPS) (33) with all participants to confirm the absence of a PTSD diagnosis. Additional exclusion criteria for this study included: a current Glasgow Coma Scale score less than 13; a history of head injury resulting in loss of consciousness for 60 min or more; active suicidal or homicidal ideation; pregnancy; a diagnosis of postconcussive syndrome (PCS) according to International Classification of Diseases, 10th Clinical Modification criteria.

The data analyzed here are from a larger study exploring predictors for postdeployment delayed-onset PTSD, major depressive disorder and PCS. Therefore, the military service members who met the diagnostic criteria for PTSD, depression or PCS at the time of recruitment were excluded from the study. The experimental protocols were approved by institutional review boards at Uniformed Services University, Walter Reed National Military Medical Center, and the National Institutes of Health. All participants provided and signed written informed consent prior to participation.

### EEG Recording and Preprocessing

Scalp EEG was recorded from all participants during eyes-closed resting state for about 2.5 min in an acoustically and electrically shielded room. The data were acquired using the EPA6 amplifier (Sensorium Inc.) and Grass electrodes (Natus Neurology Inc.) at Fz, Cz, Pz, Oz, C3, and C4 positioned according to the standard 10–20 electrode system, with linked earlobes as reference and a forehead ground. A limited number of electrodes was used to test the utility of a reduced montage that could be implemented in a far forward military medical environment. Electrode impedances were maintained under 5 kΩ. EOG was recorded from two electrodes placed below and above the right eye. The sampling rate was 2,048 Hz.

The EEG data were preprocessed offline using custom scripts written in MATLAB (www.mathworks.com). All data were first visually inspected to ensure that participants were awake throughout the recording period. Data from Oz channel were found to be contaminated by artifacts in three participants, and therefore excluded from further analysis. All the following analyses were conducted for Fz, Cz, Pz, C3, and C4 channels. EOG artifacts were corrected by using a regression approach (34). The data after EOG correction were high-pass filtered at 0.5 Hz, low-pass filtered at 50 Hz, and down-sampled to 256 Hz. To remove data portions contaminated by artifacts and to construct multiple trials for computing WPLI, we segmented the 2.5-min continuous EEG data into 1-s short epochs, resulting in 150 pseudotrials. Trials with activity exceeding 75 μV were excluded from analysis. The mean trial rejection rate was 2.83%.

### Weighted Phase Lag Index

As described by Vinck et al. (28), WPLI is a measure of phase synchronization based solely on the imaginary component of the cross-spectrum. Let *X*_1_(*f*) and *X*_2_(*f*) be the Fourier transform of the signals *x*_1_(*t*) and *x*_2_(*t*) from two separate sensors, respectively, at frequency *f*. Then the cross-spectrum between *x*_1_(*t*) and *x*_2_(*t*) is computed as

(1) $$S(f)=X_{1}(f)X_{2}^{\text{*}}(f)\text{,}$$

where $X_{2}^{\text{*}}$ is the complex conjugate of *X*_2_. If we write the Fourier transformed signals as *X*_1_(*f*) = *r*_1_exp(*i*θ_1_) and *X*_2_(*f*) = *r*_2_exp(*i*θ_2_) then the cross-spectrum can be rewritten as

(2) $$S\left(f\right)=r_{1}r_{2}\text{exp}\left(i\Delta \theta \right)=r_{1}r_{2}\text{cos}\left(\Delta \theta \right)+i\left(r_{1}r_{2}\text{sin}\left(\Delta \theta \right)\right),$$

where Δθ = θ_1_ − θ_2_ is the phase difference between the two signals at frequency *f*. In what follows, we drop inclusion of *f* for notational convenience, but note that all values are frequency dependent. Let Im(*S*) denote the imaginary part of the cross-spectrum:

(3) $$\text{Im (}S\text{)}=r_{1}r_{2}\text{sin}\left(\Delta \theta \right).$$

Then the WPLI is defined as

(4) $$\text{WPLI}=\frac{\left|\langle \text{Im(}S\text{)}\rangle \right|}{\langle |\text{Im(}S\text{)|}\rangle }=\frac{\left|\langle |\text{Im(}S\text{)}|\text{sign}(\text{Im(}S\text{))}\rangle \right|}{\langle |\text{Im(}S\text{)|}\rangle },$$

where 〈⋅〉 denotes an expectation. In practice the expectation is estimated by averaging over a large number of epochs. In Eq. 3, we see that the imaginary part of the cross-spectrum is only sensitive to synchronizations of two signals which have phase delays. From the quasistatic approximation, volume conduction does not induce a phase delay; therefore WPLI is insensitive to spurious connectivity created by volume conduction.

Weighted phase lag index has been proposed as an improved measure of phase synchronization compared to PLI (26), which is defined as

(5) PLI=|〈sign(Im(S))〉|.

From the Eq. 3, we can see that the sign of the imaginary part of the cross-spectrum indicates whether signal *x*_1_(*t*) tends to phase lead or lag signal *x*_2_(*t*). Therefore PLI estimates to what extent the phase leads and lags between two signals are nonequiprobable. Compared to PLI, WPLI weights the contribution of the observed phase leads and lags by the magnitude of the imaginary part of cross-spectrum (see the Eq. 4). Cross-spectra around the real axis contribute to a lesser extent than cross-spectra around the imaginary axis (28). This makes WPLI less sensitive to noise as cross-spectra around the real axis are at risk of changing their true sign with small noise perturbations.

The estimate of WPLI obtained from Eq. 4 has a positive bias. To solve this problem, Vinck et al. (28) propose a debiased estimator of the squared WPLI, which is defined as

(6) $$\text{debiased WPLI-square}=\frac{\sum_{j=1}^{N}\sum_{k\neq j}\text{Im}\left(S_{j}\right)\text{Im}\left(S_{k}\right)}{\sum_{j=1}^{N}\sum_{k\neq j}\left|\text{Im}\left({\text{S}}_{\text{j}}\right)\text{Im}\left({\text{S}}_{\text{k}}\right)\right|}\text{,}$$

where *N* is the number of epochs and *S_j_* and *S_k_* are the cross-spectrum between a pair of channels for the *j*-th and *k*-th epochs, respectively. In this study *N* was typically about 150. The smallest value of *N* used was 133. Equation 6 is debiased (strictly asymptotically unbiased) because it converges to the square of Eq. 4 in the limit of an infinite number of epochs. The theoretical development of this debiased estimator and its statistical performance can be found in the original method by Vinck et al. (28).

In the current study, we used the debiased WPLI-square estimator as the measure of EEG phase synchronization. We computed the debiased WPLI-square (which will be referred to as WPLI throughout the rest of the article) for all pairs of EEG electrodes based on Eq. 6. Cross-spectra were computed using the Fourier transforms of the preprocessed 1-s data epochs. The frequency resolution thus equaled 1 Hz.

### DTI Acquisition and Processing

Twelve out of 15 mTBI cases and 17 out of 22 controls had their DTI data recorded within a two day period from the EEG recording. DTI data were acquired on a Siemens Biograph mMR 3 T scanner with parameters TR (repetition time) = 17,000 ms, TE (echo time) = 98 ms, flip angle = 90°, voxel size = 2 mm × 2 mm × 2 mm, matrix size = 128 × 128, and slices = 75. The acquisition included 10 images at *b* = 0 s/mm^2^, 10 images with non-collinear directional gradients at *b* = 300 s/mm^2^, and 60 images with non-collinear directional gradients at *b* = 1,100 s/mm^2^. Images were processed using the CATNAP software for tensor estimation (35). Two *b*-values were included for more robust tensor estimation (36) as well as for potentially exploring more sophisticated diffusion models in the future. All diffusion weighted images were included in the single tensor fit. Images were preprocessed for motion correction and eddy current correction, with adjustments to the gradient table performed based on patient position. Distortions due to echo planar imaging susceptibility artifacts were corrected by performing a deformable registration to an anatomic T2-weighted acquisition (TR = 3,200 ms, TE = 409 ms, flip angle = 120°, voxel size = 1 mm × 1 mm × 1 mm, matrix size = 256 × 256, slices = 176). Following preprocessing, all images were used to perform tensor estimation. Next, automated segmentation of white matter tracts was performed using the Diffusion-Oriented Tract Segmentation (DOTS) algorithm (37). DOTS combines statistical atlases of tract location and tensor orientation with a Markov random field model to map diffusion properties to tract labels. This enables average values of fractional anisotropy (FA) to be computed for each labeled tract. The statistical region of interest (ROI) atlas was based on the atlas of Ref. (38). We included the following fiber tracts which are susceptible to mTBI (39–43): corpus callosum, inferior and superior longitudinal and fronto-occipital fasciculi, cingulum, fornix, uncinate fasciculus, optic radiation, corticospinal and corticopontine tracts, inferior, middle, and superior cerebellar peduncles (separated for left and right when applicable).

### Statistical Analyses

Differences between groups in demographics and psychological measures were examined by two-sample *t*-tests whether the data are numerical or Fisher’s exact tests whether the data are categorical. The comparisons between the mTBI and control groups for the WPLI measures were tested using the Wilcoxon rank-sum tests. In order to identify oscillatory frequencies in neural synchronization showing abnormality following mTBI, we first performed the tests on the theta (4–7 Hz), alpha (8–13 Hz), beta (15–23 Hz), and low-gamma (25–40 Hz) frequency bands for WPLI spectra averaged across all electrode pairs. Then for the identified frequency band, we further performed the tests for each electrode pair to reveal the spatial pattern of the abnormality. To test whether or not the estimated WPLI values are significantly larger than zero, an empirical distribution technique using surrogate data was applied (44, 45). Specifically, we randomly and independently shuffled the time series data from each electrode to create a surrogate data set. Then the WPLI measures were computed from the surrogate data set. After repeating this process 1,000 times, we created empirical distributions for the WPLI measures. Since the shuffling process destroys all the temporal structure in the data, the empirical distributions provide variability for the null hypothesis case. We then used these distributions to assess the significance of the WPLI measures estimated from the actual data. For the FA measures from the DTI data, the comparisons between mTBI and control groups were tested using two-sample *t*-tests for each ROI and the *p*-values were corrected for multiple comparisons using the Holm-Bonferroni method. To examine the association between disruption of neural synchronization and the abnormality of white matter integrity, we computed Pearson’s correlation coefficient across participants between the WPLI measures and the FA measures that are found statistically significant between groups. For the WPLI measure, in order to accommodate the individual differences for the null hypothesis case, the measure of each participant was first normalized by its empirical null distribution (subtracted the mean and divided by the standard deviation) and then correlated with FA measure across participants. Statistical significance of correlation coefficients was tested by a *t*-statistic created from Fisher transform. The *p*-values less than 0.05 were considered statistically significant.

## Results

### Participant Characteristics and Psychological Measures

Table 1 summarizes the participant characteristics and psychological measures. Age, gender, and handedness were found to be statistically indistinguishable between the mTBI and the control groups (*p* > 0.05). Compared to controls, mTBI cases showed significantly higher PTSD scores including CAPS (total score, Criterion B subscore for Reexperiencing, Criterion D subscore for Hyperarousal) and PCL-M scores (*p* < 0.05). The PHQ-9 score for depression was also higher for mTBI compared to controls, but the difference did not reach significance (*p* = 0.076). The PSQI score for sleep quality over the past month was not statistically different between the two groups (*p* > 0.05).

**Table 1:** Participant characteristics and psychological measures.

Variable	mTBI (*n* = 15)		Controls (*n* = 22)^a^		Group comparison^b^		
	Mean	SD	Mean	SD	*df*	*t*-Value	*p*-Value
Age	27.1	6.2	27.9	6.1	35	0.38	0.71
Gender, male/female	12/3		21/1				0.15
Handedness, R/L	13/2		19/3				0.37
CAPS total	25.0	13.6	15.7	10.8	35	−2.32	0.027*
CAPS Criterion B	6.9	5.1	2.6	3.1	35	−3.17	0.0031*
CAPS Criterion C	5.1	4.1	3.8	5.6	35	−0.80	0.43
CAPS Criterion D	13.0	6.8	9.3	4.2	35	−2.07	0.046*
PSQI score	6.9	3.1	6.3	2.5	33	−0.66	0.52
PHQ-9 score	3.1	1.9	1.9	2.1	34	−1.83	0.076
PCL-M score	32.1	7.8	23.9	6.9	34	−3.37	0.0019*

*^a^n = 21 for PHQ-9 score and PCL-M score; n = 20 for PSQI score*.

*^b^Fisher’s exact tests were used for gender and handedness. Student’s t-tests were used for other variables*.

*p < 0.05.

### EEG Phase Synchronization between Brain Regions

**Figure 1:**
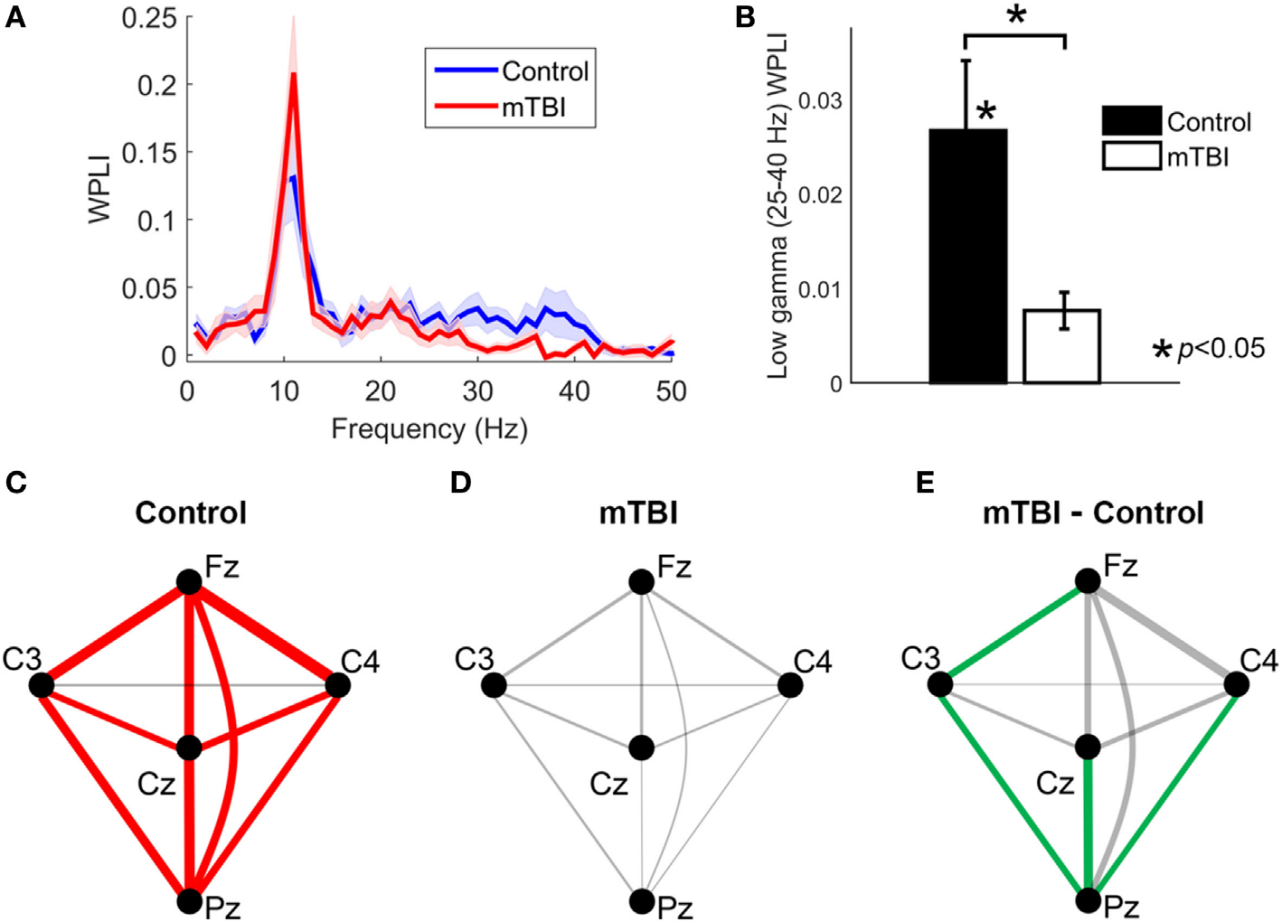
The analysis of weighted phase lag index (WPLI) for control and mild traumatic brain injury (mTBI) groups. **(A)** The WPLI spectra averaged across all electrode pairs. Shaded areas indicate the standard error of mean across participants at each frequency. **(B)** A bar plot comparing the mean value of WPLI spectra within low-gamma frequency (25–40 Hz) between control and mTBI groups averaged over all electrode pairs. **(C,D)** Low-gamma phase synchrony (measured by WPLI) for different electrode pairs for the control and mTBI groups, respectively. Line thickness is proportional to synchronization strength. Significant connections (*p* < 0.05) are indicated by red lines. **(E)** The difference of low-gamma phase synchrony between mTBI and control groups for different electrode pairs. The electrode pairs with significant smaller low-gamma phase synchrony in mTBI group compared to control group (*p* < 0.05) are indicated by green lines.

Using the WPLI spectra averaged across all electrode pairs, comparisons between the control and mTBI groups revealed that the mean WPLI value in low-gamma frequency band (25–40 Hz) was significantly smaller (*p* = 0.0082) in those with a history of mTBI (Figures 1A,B). Moreover, application of testing using surrogate data found that the mean WPLI value in the low-gamma frequency band was significantly larger than zero for the control group (*p* < 0.001), but not significantly different from zero for the mTBI group (*p* > 0.05). The mean WPLI values in theta (4–7 Hz), alpha (8–13 Hz), and beta (15–23 Hz) frequency bands were not significantly different between two groups (*p* > 0.05).

The phase synchronization (measured by WPLI) at low-gamma frequency for different electrode pairs are displayed in Figures 1C,D for the control and the mTBI groups, respectively. The synchronization strength is indicated by line thickness and the significance of synchronization is indicated by red color. For the control population all pairs of electrodes except the C3-C4 pair exhibited significant (that is, greater synchronization than observed with surrogate data) low-gamma synchronization (Figure 1C). In contrast, none of the electrode pairs showed significant low-gamma synchronization for the mTBI cases (Figure 1D), suggesting a global reduction of synchronization instead of a reduction for only one or two electrode pairs. By comparing the two groups, we revealed that the reduction of low-gamma synchronization in the mTBI group was significant for the Cz-Pz (*p* = 0.0042, uncorrected), Fz-C3 (*p* = 0.023, uncorrected), C3-Pz (*p* = 0.017, uncorrected), and C4-Pz (*p* = 0.017, uncorrected) electrode pairs (see Figure 1E, significant electrode pairs were indicated by lines in green color).

### DTI White Matter Integrity

**Figure 2:**
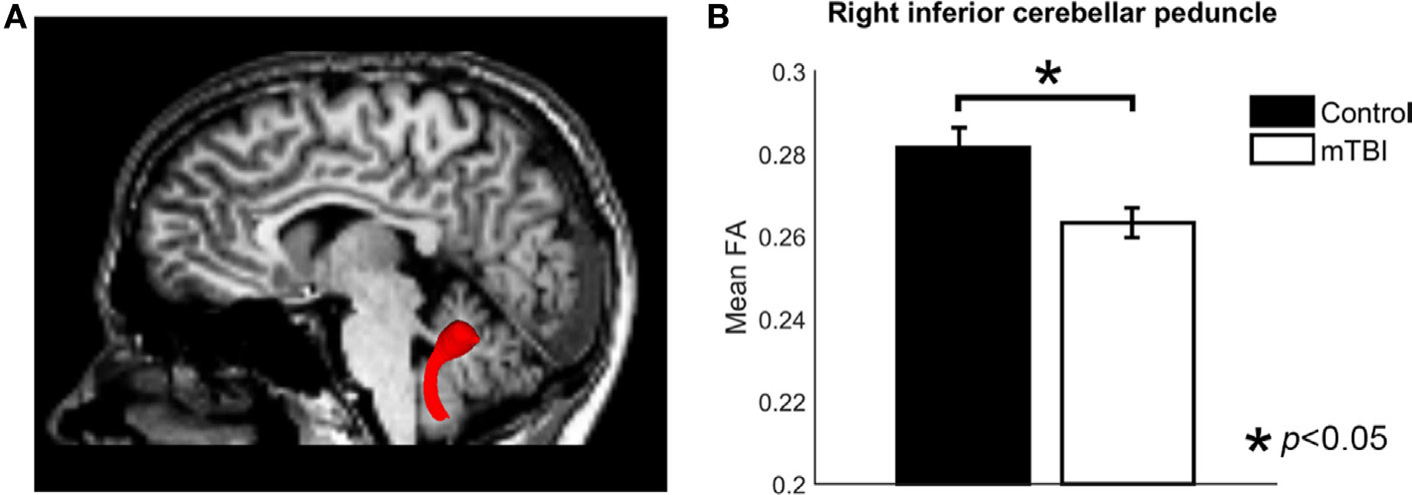
The analysis of fractional anisotropy (FA) for control and mild traumatic brain injury (mTBI) groups. **(A)** Image showing the inferior cerebellar peduncle region of interest. **(B)** A bar plot comparing the mean FA value of right ICP between control and mTBI groups.

Region of interest-based comparison revealed reduced FA in the white matter of the right inferior cerebellar peduncle (*p* = 0.0015, uncorrected, after correction *p* = 0.042) in those with a history of mTBI (Figure 2). The left inferior cerebellar peduncle ROI also showed a reduction of FA but did not reach significance after correction (*p* = 0.026, uncorrected, after correction *p* > 0.05). No significant differences were found for other ROIs (*p* > 0.05, uncorrected). The comparison results for all ROIs are shown in Table 2.

**Table 2:** Group results for fractional anisotropy (FA) for each ROI.

ROI	Side	Fractional anisotropy				*p*-Value
		mTBI (*n* = 12)		Controls (*n* = 17)		
		Mean	SD	Mean	SD	
Corpus callosum	Front	0.377	0.009	0.361	0.011	0.31
	Posterior	0.422	0.005	0.415	0.008	0.49
	Superior	0.420	0.006	0.412	0.008	0.44

Cingulum	Left	0.345	0.009	0.337	0.007	0.46
	Right	0.349	0.006	0.345	0.006	0.71

Corticopontine tract	Left	0.419	0.007	0.412	0.009	0.58
	Right	0.417	0.006	0.417	0.008	0.96

Corticospinal tract	Left	0.432	0.012	0.422	0.011	0.53
	Right	0.408	0.009	0.408	0.009	0.96

Fronix	Left	0.294	0.004	0.296	0.007	0.86
	Right	0.297	0.006	0.300	0.006	0.92

Inferior cerebellar peduncle	Left	0.273	0.004	0.289	0.005	0.026*
	Right	0.265	0.003	0.285	0.004	0.0015**

Inferior fronto-occipital fasciculus	Left	0.341	0.005	0.339	0.008	0.77
	Right	0.344	0.006	0.341	0.007	0.76

Inferior longitudinal fasciculus	Left	0.361	0.007	0.358	0.009	0.85
	Right	0.353	0.006	0.349	0.009	0.76

Middle cerebellar peduncle		0.334	0.005	0.332	0.004	0.78

Optic radiation	Left	0.399	0.007	0.392	0.010	0.60
	Right	0.378	0.007	0.377	0.011	0.96

Superior cerebellar peduncle	Left	0.322	0.007	0.331	0.008	0.42
	Right	0.289	0.005	0.303	0.007	0.17

Superior fronto-occipital fasciculus	Left	0.357	0.006	0.351	0.008	0.59
	Right	0.344	0.007	0.343	0.007	0.94

Superior longitudinal fasciculus	Left	0.375	0.004	0.368	0.009	0.53
	Right	0.360	0.005	0.355	0.008	0.65

Uncinate fasciculus	Left	0.279	0.011	0.281	0.009	0.89
	Right	0.291	0.007	0.301	0.009	0.43

*p < 0.05 before correction.

**p < 0.05 after correction.

### Correlation between EEG Phase Synchronization and White Matter Integrity

**Figure 3:**
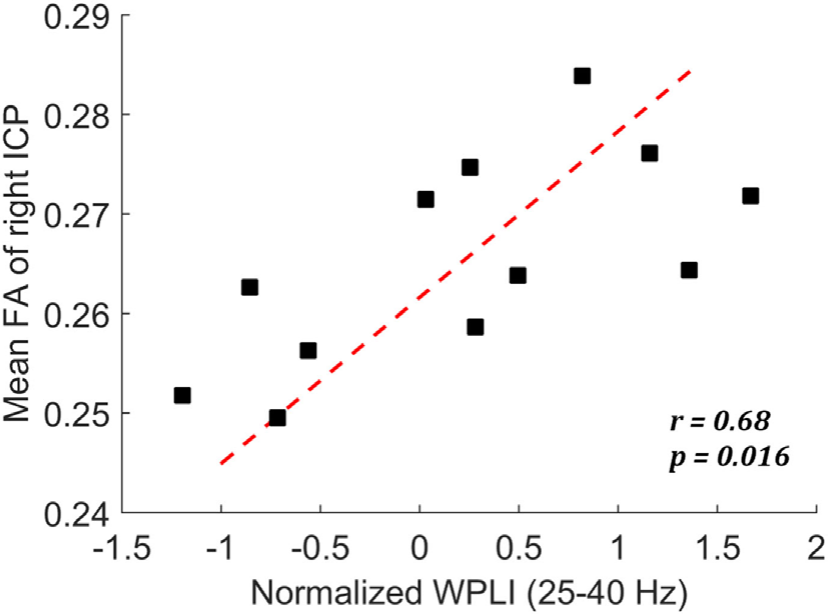
Scatter plot showing the correlation between mean fractional anisotropy (FA) of right ICP and the normalized weighted phase lag index (WPLI) at low-gamma frequency among the mild traumatic brain injury cases.

We have identified reduced low-gamma phase synchronization from the EEG data and reduced white matter integrity of the right inferior cerebellar peduncle from the DTI data for those with a history of mTBI. The correlation analysis further revealed that, among mTBI cases, these two measures were significantly correlated (*r* = 0.68, *p* = 0.016) (Figure 3). Weaker low-gamma phase synchronization (averaged across all electrode pairs) corresponded to smaller FA value of the right inferior cerebellar peduncle. Conversely, for controls there was no significant correlation between the two measures (*p* > 0.05).

### Correlation between Physiological and Psychological Measures

Exploration of the relationship between low-gamma phase synchronization and psychological measures (CAPS, PCL-M, PHQ-9, and PSQI) yielded no significant correlations for either group (*p* > 0.05). Higher low-gamma phase synchronization tends to associate with lower CAPS Criterion D subscore for Hyperarousal in mTBI group, but the correlation (*r* = −0.43, *p* = 0.11) was not statistically significant. Exploration of the relationship between the FA value of the right inferior cerebellar peduncle and the psychological measures also yielded no significant correlations for either group (*p* > 0.05).

## Discussion

In the present study, we investigated the impact of mTBI on the intrinsic synchronization of EEG activity and examined the association between the disruption of EEG phase synchronization and the disruption of white matter integrity in a cohort of military service members. Applying debiased WPLI, a newly developed measure of neural synchronization which is less sensitive to the effects of volume conduction, we found that the service members with a history of mTBI exhibited diminished EEG phase synchrony at low-gamma frequency (25–40 Hz) across scalp regions relative to the controls suggesting disrupted functional coordination between brain regions as a consequence of mTBI. Moreover, we found that for the mTBI cases, the diminished low-gamma phase synchrony was significantly correlated with the white matter integrity (FA) of the inferior cerebellar peduncle, which was also found significantly diminished in the mTBI cases relative to the controls. These findings yield evidence for a correlation between the impairment of white matter integrity and the impairment of functional synchronization in the brain after mTBI.

Phase synchronization of neural oscillations is widely acknowledged as a fundamental mechanism that coordinates distributed processing of neuronal groups (16, 46–48). Several studies have reported diminished phase synchronization in mTBI patients (22–24), suggesting inefficient communication between brain regions due to head injury. Particularly, one study (23) in soldiers after blast-related mTBI identified diminished frontal phase synchrony in various frequency bands and found that phase synchrony in high frequency (beta and gamma) bands were associated with the integrity of white matter tracts of the frontal lobe. To the best of our knowledge, this is the only other existing study that has examined the relationship between phase synchronization and white matter integrity of the human brain after mTBI. Although these initial findings were intriguing and provided insight to the structural-functional connection, the results of that study are limited in several ways. First, the study used PLV which is prone to the effects of volume conduction when computing EEG phase synchrony. Second, the study did not reveal any deficiency of the white matter tracts in blast injured group which may be due to their small sample size (nine mTBI and eight controls). Third, the controls were from a civilian population, and therefore may not represent valid controls for a military population. Our study advanced this line of research by (1) performing a nested case–control comparison with a larger sample size, (2) applying a measure of phase synchronization robust to volume conduction, and (3) showing a correlation between disrupted neural synchronization and deficient axonal connection in the human brain after mTBI.

Synchronization in the gamma frequency is mostly viewed as supporting local communications within one cortical area (16, 49, 50); however, long-range gamma coupling across widely separated brain regions has been observed in attention (51), learning (52), and conscious perception (53, 54), suggesting that it plays a role in large-scale neural coordination, perhaps reflecting the synchronization of neural assemblies involved in integration of sensory processing and sensory-motor coordination (55, 56). The mechanism by which long-range gamma synchronization is generated remains unclear but is likely to involve interneuron networks. Diminished global gamma synchronization has been posited as a key feature in schizophrenia (57, 58) where the integration of sensory input with stored information is disturbed. These pieces of evidence suggest that the disrupted gamma synchronization for the mTBI group may indicate a suboptimal brain function of integration due to head injury.

The finding of reduced anisotropy of the inferior cerebellar peduncle is consistent with the notion that axonal injury is a major neuropathological consequence of mTBI. Cerebellar damage is often seen after TBI even when the initial injury does not directly involve this structure (59). Injury to cerebellar white matter has been characterized in both animal models and human studies of mild brain trauma (60–63). Specifically, studies of blast-related mild TBI in military personnel (64, 65) consistently reported reduction in anisotropy of the cerebellar peduncle. The evidence suggests that the cerebellar axonal pathways may be particularly vulnerable to blast exposure, which is the type of injury most common in our study population.

The correlation between gamma synchronization and anisotropy of the inferior cerebellar peduncle in the mTBI group suggests that the disruption of gamma synchronization may be the functional expression of the cerebellar white matter pathology. Cerebellar inactivation has been shown to disrupt the gamma coherence between the sensory and the motor cortices in rats during free whisking (a rhythmic forward and backward motion of the whiskers) (66), providing direct evidence for the cerebellar control of cortical gamma coupling. The inferior cerebellar peduncle, although contains mainly fibers entering the cerebellum, it also carries information leaving the cerebellum to the vestibular nuclei in the brainstem (67), which plays an important role in modulating the coherent activity in corticothalamic networks (68). It is possible that the deficiency of the inferior cerebellar white matter affects the integrity of the cortical–thalamic–cerebellar circuitry and thereby leads to a global disruption of gamma synchronization. Studies in schizophrenia provide indirect support for this hypothesis. Dysfunction in the cortical–thalamic–cerebellar circuitry (69, 70) and disruption in gamma synchronization (57, 58) have both been recognized as key features of impaired coordination and integration of mental processes in schizophrenia, suggesting a potential linkage between the two. Yet, it is also possible that the identified correlation reflects only covariation of the two measures due to the influences from common factors rather than a direct link. Nevertheless, the correlation demonstrated here is an important first step toward linking anatomical and functional pathophysiology following mTBI.

Analyses of possible confounding factors in the study yield no evidence that factors other than head injury explained the diminished gamma synchronization. The mTBI and controls are all military service members with similar age, gender, handedness, and deployment experience, suggesting that the findings are unlikely due to demographic influence. The sleep quality was also comparable between the two groups and thus ruled out potential sleep confounds. The psychological measures of PTSD and depression were found elevated in the mTBI group; however, the correlations with gamma synchronization were not significant, indicating that the diminished gamma phase synchrony is unlikely mediated by psychological stress. One limitation of the study is that we did not perform neuropsychological tests for assessing cognitive functions. It is therefore unclear whether the diminished gamma synchronization would correlate with any cognitive impairment. Yet, linking synchronization measures to neuropsychological performance has previously proven challenging (23). It is possible that the brain has incorporated certain coping mechanisms for the damage (71), leading to the absence of cognitive impairments despite the abnormalities in neural synchronization and axonal connection.

There is a great need for enhancement in outcome measures associated with mTBI, as the “gold standard” diagnostic criteria for TBI have not yet been established (72). Specifically, for mild TBI, conventional imaging modalities such as CT and MRI frequently failed to show any difference between head-injured patients and healthy individuals, even with patients experiencing persistent cognitive symptoms (73). DTI is a promising method for characterizing microstructural changes in mTBI, but such advanced technique is not ubiquitously available. In contrast, EEG technology is ubiquitous, portable and has been ruggedized for use in far forward clinical environments, and thus offers the potential for significant operational advantages over imaging technology. Our findings of diminished EEG gamma synchronization and its correlation with white matter abnormality in mTBI may prove useful in improving the diagnostic, monitoring, and treatment capabilities for mTBI. Caution must be exercised in the interpretation of these results. The results presented here do not demonstrate that gamma synchronization abnormalities will necessarily provide a successful diagnostic biomarker for mTBI, because statistically significant group separations do not ensure success as a classifier (74). The clinical aspects of the measure, such as the diagnostic sensitivity and specificity and the association with recovery and treatment, and the comparison of the utility of WPLI versus the conventional synchronization measures, require further study.

Significant limitations of this study should be noted explicitly. Because the injuries were sustained in theater, there was a considerable heterogeneity in both the type of injury and the time from injury to evaluation. Additionally, the number of electrode sites is limited. Future studies using high-density EEG/MEG with full scalp coverage are desired. Moreover, the sample size was small, largely restricted to males, and the results do not have the statistical power to identify gamma synchronization as a diagnostic biomarker for mTBI.

## Conclusion

Both mTBI and controls were from the same cohort of military service members and were evaluated within two months of their return from a deployment in either Iraq or Afghanistan. Synchronization at low-gamma frequency, as quantified by the WPLI, was significantly decreased in the mTBI cases compared against controls, and the disrupted low-gamma synchronization was significantly correlated with the white matter integrity of the inferior cerebellar peduncle which was also significantly reduced in the mTBI group. These findings yield evidence for a correlation between the impairment of white matter integrity and the impairment of functional synchronization in the brain after mTBI.

## Ethics Statement

The experimental protocols were approved by institutional review boards at Uniformed Services University, Walter Reed National Military Medical Center, and the National Institutes of Health. All subjects provided and signed written informed consent prior to participation.

## Author Contributions

CW performed the electrophysiological data analysis, statistical analysis, and wrote the drafts of the article. MC screened participants for eligibility, conducted the psychological assessments, and obtained diffusion tensor imaging data. PR and DD participated in developing the statistical analysis plan and writing the drafts. DN and KB obtained the electrophysiological data. DP performed the analysis of the diffusion tensor imaging data. MR participated in the design of the investigation. DK lead the research effort and participated in acquisition of the electrophysiological data.

## Conflict of Interest Statement

The authors declare that the research was conducted in the absence of any commercial or financial relationships that could be construed as a potential conflict of interest.
